# Motor Imagery Development in Children: Changes in Speed and Accuracy With Increasing Age

**DOI:** 10.3389/fped.2020.00100

**Published:** 2020-03-13

**Authors:** Deisiane Oliveira Souto, Thalita Karla Flores Cruz, Patrícia Lemos Bueno Fontes, Rodrigo Caetano Batista, Vitor Geraldi Haase

**Affiliations:** ^1^Graduate Program in Neurosciences, Federal University of Minas Gerais, Belo Horizonte, Brazil; ^2^Developmental Neuropsychology Laboratory, Department of Psychology, Universidade Federal de Minas Gerais, Belo Horizonte, Brazil; ^3^Department of Physiotherapy, Pontifícia Universidade Católica de Minas Gerais, Betim, Brazil; ^4^National Institute of Science and Technology on Behavior, CNPq, Belo Horizonte, Brazil

**Keywords:** motor imagery, development, children, adults, mental rotation

## Abstract

Although motor imagery has been pointed as a promising strategy for the rehabilitation of children with neurological disorders, information on their development throughout childhood and adolescence is still scarce. For instance, it is still unclear at what age they reach a development comparable to the motor imagery performance observed in adults. Herein we used a mental rotation task to assess motor imagery in 164 typically developing children and adolescents, which were divided into four age groups (6–7, 8–9, 10–11, and 12–13 years) and 30 adults. The effects of biomechanical constraints, accuracy, and reaction time of the mental rotation task were considered. ANOVA showed that all groups had the effect of biomechanical restrictions of the mental rotation task. We found a group effect for accuracy [*F*_(4, 180)_ = 17,560; *p* < 0.00; η^2^ = 3.79] and reaction time [*F*_(4, 180)_ = 17.5; *p* < 0.001, η^2^ = 0.615], with the results of children groups 6–7 and 8–9 years being significantly lower than the other groups (*p* < 0.05). In all the analyses, there were no differences regarding accuracy and reaction time among the participants of the age groups 10–11 and 12–13 years and adults (*p* > 0.05). Concluding, children aged 6–7 years were able to perform motor imagery, motor imagery ability improved as the participants' ages increased, and children aged 10 and over-performed similarly to adults.

## Introduction

The ability to mentally simulate actions without physically performing them is one of the most remarkable skills of the human mind. Motor Imagery (MI) can be defined as a dynamic cognitive process in which an individual mentally simulates an action without the external manifestation of the motor act (1, 2). According to Jeannerod (3) MI is the representation of the action involved in the planning and execution of the movements. Mental simulation of movement is important because it follows the intentions and plans of motor acts, assessing whether the actions performed correspond to the desired actions (3, 4). Thus, MI exhibits many of the properties of motor planning and is considered a valid method for training the internal action control model (5). The internal motor control model proposed by Wolpert (4) is a neural system that simulates the next action. This model acts as a predictor in the central nervous system, providing predictions that allow the planning and successful execution of the action (4, 6). Thus, for each intended action, the nervous system issues a motor command to the muscles, while a copy of the motor command is used to predict the future state of the moving limb (6, 7).

According to Jeannerod (8), imagined movements are functionally equivalent to those performed physically in terms of intentions, motor planning, and motor program engagement. In fact, functional neuroimaging studies have shown that MI activates a set of neural networks (parietal, frontal motor, and cerebellar areas) that partially overlap the brain network that is activated during motor performance (9–12). Thus, as MI and motor execution are closely related processes, MI is increasingly being explored to improve motor skill acquisition by stimulating the neural networks underlying movement planning and control (2, 13, 14). Indeed, improvements in the performance of motor skills associated with MI training have been documented in healthy people (15, 16) and in clinical populations, particularly in post-stroke patients (17). Specifically, repetitive activation of neural pathways during MI activates the neuroplasticity mechanisms underlying motor learning, providing a rationale for their use in neuro-rehabilitation. Therapy based on MI and interventions based on the physical practice induce brain plasticity required for functional recovery (18).

To improve motor skills, individuals must imagine all the sensations that accompany the physical performance of the imagined task (19). Therefore, determining the extent to which images are used by an individual is critical to ensure the success of the intervention. A variety of MI measurements are available. The vast majority of research involving children uses the mental rotation task or mental chronometry to assess MI ability (20–23). The present study focuses on the investigation of the capacity of MI using exclusively the task of mental rotation.

Studies that applied the task of mental rotation associated with neuroimaging observe a significant motor activation of the cortex when participants imagined the mental rotation of the hand figures (23). In a recent study involving transcranial magnetic stimulation (TMS), Hyde et al. (24) suggest that the motor cortex is activated during the performance of HLJ. In this task, hand figures are presented in different spatial orientations and individuals mentally simulate the movements of their own hands and decide whether the figures represent the left or right hands. The linear relationship between the angle of rotation and reaction times (RT) proposed by Parsons (25) was confirmed by studies showing that biomechanical constraints that apply to physical motion also restrict imagined motion (21). The effect of biomechanical constraints refers to increase in RTs when hand figures are presented in anatomical positions that make mental rotation difficult (Figures with fingers facing sideways). Similarly, a decrease in response time is observed when the stimuli are medially rotated (figures with fingers facing medially). The presence of the effect of biomechanical constraints on the task confirms that individuals indeed used MI (1, 26). de Lange et al. (27) evaluated brain activation of healthy individuals while performing the mental rotation task using functional magnetic resonance and found stronger activation of pre-motor and intraparietal regions when individuals responded to stimuli presented in medial positions when compared to lateral stimuli. These findings show that there are indeed differences in judging hand images in medial and lateral postures, therefore providing further support for the hypothesis of the effect of biomechanical constraints.

In addition to changes in RT as a function of the rotation angle, there is a postural effect of the mental rotation task that strengthens the presence of the effects of biomechanical constraints. Thus, the position of the participant's body during the task may influence the recognition of hand laterality (19, 25, 28). This is because the volunteer simulates the movement of one's body from its current position, and not from a fixed representation in the brain (27). To solve the task, the individual keeps his/her hand in the back posture, and therefore shorter RTs for stimuli in this posture are expected than RTs for stimuli presented in the palm view.

Studies involving the adult population established that at this age there is a complete maturation of the mechanisms involved in MI (29). However, there is great controversy as to the minimum age when a child is able to engage in tasks using MI (1, 20, 22, 30, 31). Moreover, the age when they reach development comparable to that observed in adults remains unclear. According to Funk et al. (32), there are few studies investigating the development of MI. In addition, from studies evaluating MI in children, most compared typically developing children to those with Cerebral Palsy or Development Coordination Disorder—DCD (33–37).

From studies that evaluated MI in children using variations of the mental rotation task, some reported the presence of the effect of biomechanical restrictions for children between 5 and 12 years of age (20, 21, 32, 38), suggesting that in this age group they are already capable of performing MI based on motor processes. In the study by Funk et al. (32), about 60% of children aged 5 to 6 years were able to use MI, compared with 100% of adults. However, in a later study, Butson et al. (22) state that most children aged 5 and 6 years were unable to perform the task accurately above 50% of the correct level. Furthermore, these authors confirmed the presence of the effect of biomechanical restrictions only in children aged 8, 9, and 11 years, in children aged 7 and 10 years, this effect was not found. There is still controversy regarding changes in the effect of biomechanical constraints as age increases. In the study by Funk et al. (32) the impact of biomechanical constraints and hand posture on solving the mental rotation task was greater in children than in adults, suggesting that children are even more guided by motor processes than the adults. In contrast, this claim was challenged by a later study showing that biomechanical constraints were stronger in 8-year-olds than in 6-year-olds (39).

Caeyenberghs et al. (21) compared performance in the mental rotation task of 7- and 8-year-olds, 9- and 10-year-olds, and 11- and 12-year-olds and found that younger children (7 and 8-year-olds) are generally less accurate and slower than older children (11 and 12 years). This finding suggests that there are progressive improvements in MI skills as age increases. In a more recent study, Fuelscher et al. (38) point to a non-linear relationship between the MI ability and age in the HLJ task. These authors also stated that, in these children from 6 to 12 years old, MI ability is associated with motor planning ability, since they are closely related processes. However, the authors are cautious in interpreting these results in view of the modest sample size.

Taken together, studies of age-related differences in MI indicate that children's ability to accurately perform the mental rotation task increases with age. However, the literature review by Spruijt et al. (20) suggests that it is not possible to draw definitive conclusions from studies using the mental hand rotation task on the exact development of MI in children. Given the small sample size of the studies, sample error is a major concern and probably contributed to the controversial group comparisons reported in previous studies. Moreover, the limited age ranges proposed by the studies do not allow definitive conclusions about the development of MI in children, its evolution during childhood, adolescence and adulthood.

Given the controversies explicit in the literature, the temporal course of development and the underlying mechanisms have not yet been sufficiently clarified. Involving a larger sample (194 children) and a wider age range (from 6 to 13 years old) than previous studies, and using the mental rotation task herein we investigated: (a) if younger children are already able to perform MI tasks; (b) if children follow the biomechanical constraints to solve the task; (c) if there is influence of postural perspective of the hand: dorsal vs. palmar; (d) if there are age-related differences; and (e) at what age children's MI performance resembles that of healthy adults. To this end, we analyzed the effects of biomechanical constraints on RTs, the effects of back and palm visual perspectives, and the age differences for accuracy and RT.

## Materials and Methods

### Participants

The total sample consisted of 194 volunteers, of whom 164 are children (88 boys and 76 girls), recruited from a public school in southeastern Brazil (city of Betim, Minas Gerais, Brazil). The ages of the participants ranged from 6 years and 5 months to 13 years and 2 months (mean age = 9.52 ± 2.10 years). Children and adolescents were assembled into four age groups: 6–7, 8–9, 10–11, and 12–13 years old (Table 1). A group of 30 adults was also recruited in Betim, Minas Gerais, Brazil. Only right-handed individuals presenting normal or corrected vision, lack of neuromotor impairment, able to discriminate right and left were included. Before the study initiated, written consent was obtained from the adults as well as from the parents/guardians of the children and adolescents recruited. All research procedures were approved by the Research Ethics Committee of the Federal University of Minas Gerais (COEP/UFMG).

**Table 1:** Sex and age of groups.

	**Sex**		**Age**	
	**Male**	**Female**	**M**	**SD**
Group 6–7 years old (*n* = 37)	19	18	6.69	0.48
Group 8–9 years old (*n* = 40)	26	14	8.45	0.53
Group 10–11 years old (*n* = 39)	21	18	10.49	0.65
Group 12–13 years old (*n* = 34)	14	20	12.60	0.51
Group adult (*n* = 30)	13	17	25.77	1.99

*M, mean; SD, Standard deviation*.

### Measurements

#### Laterality Dominance

Lateral dominance of hand was assessed by the Laterality Task (40). The participant sat in a chair facing a table. A small ball was placed by the examiner in the center of the table. Then, the participant was instructed to take the ball with one hand and throw it into a basket that was positioned in front of the table. The test was repeated three times. The volunteer who used his right hand to catch and throw the ball in all three attempts was considered right-handed.

#### Right–Left Orientation

To evaluate right-left orientation we used the Right-Left orientation test (41). The test has 12 items of right and left body parts recognition. It is divided into three steps: the first presents simple commands regarding the child's own body, the second consists of double commands—direct and crossed—toward the child's body. In the third step, pointing commands to single lateral body parts of an opposite-facing person was issued. Correct answers were scored as one and wrong answers scored as zero.

#### Motor Imagery

**Figure 1:**
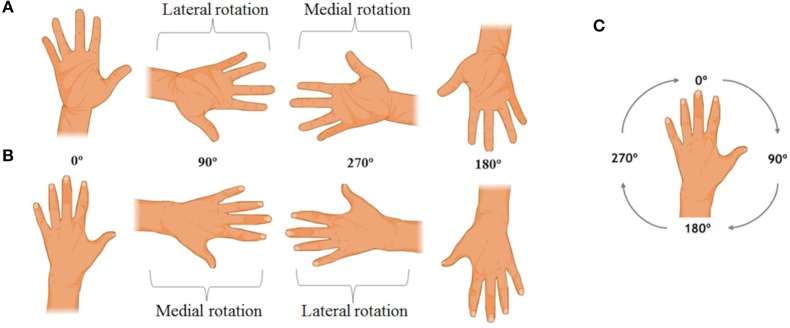
Examples of the hand laterality judgment task stimuli. In **(A)**, the right hand stimuli are observed in palm view. In **(B)**, the left hand stimuli are presented in back view, and in **(C)** the rotation direction of the task stimuli is indicated.

The ability of MI was measured by the hand laterality judgment task (HLJ; Figure 1), which is a variation of the mental rotation task (27). This is a computerized task in which, on a computer screen, figures of the hands (right and left) are presented in different views (back and palm) and rotation angles (0°, 90°, 180°, and 270°). The task consists of 16 different stimuli, repeated five times each, totaling 80 stimuli. The HLJ task evaluates the MI by requiring the individual to imagine his own hand moving to the orientation presented in the stimulus to make the laterality judgment. The use of MI to solve the HLJ task is characterized by differences in RT and accuracy as well as by the presence of the effect of biomechanical constraints (1, 25). This effect is characterized by an increase in RT as a function of the rotation angle of the stimuli (17). The stimuli in which the hand figures are medially oriented are anatomically easier to rotate mentally and therefore the resulting RT to recognize medially oriented stimuli should below. Also, judging laterality when the stimulus presented is the left hand rotated 90° (medial rotation) is faster than when the right hand at 90° is shown (25, 27).

### Procedures

The participants were positioned at a comfortable distance from the computer screen and instructed to decide as quickly and accurately as possible whether each stimulus was a left or a right hand. Hand stimuli were randomly presented at 4 different angles of rotation (using the Presentation software, version 0.71) and remained on the screen until a response was recorded by pressing a designated key on the computer keyboard. Moreover, the volunteers were instructed to imagine their own hand turning to the position of the presented stimulus and then decide if the stimulus corresponded to the right or left hand. The literature review by Spruijt et al. (20) states that it is not possible to infer whether or not to use instructions to solve the mental rotation task, due to methodological variations of the studies developed. Thus, based on previous studies (21, 22, 42) our study chose to provide instructions to participants. Participants remained with their hands in the pronated posture (back of the hand up) positioned close to the computer keyboard. Participants were prohibited from moving their hands. The volunteer was instructed to use his/her index fingers to respond by pressing the right computer key with his right finger when the picture was considered to correspond to the right hand and the left computer key when the picture was considered to correspond to the left hand. Accuracy and RT records were produced for each stimulus by and later used for data analysis.

### Data Analysis

Tests in which participants missed or produced RTs greater than three standard deviations above or below the overall average were excluded from the analyzes. The average time and precision, as well as the average time in medial and lateral rotation for the palmar and dorsal views, were calculated for every participant. To compare the means obtained for accuracy and RTs we performed analysis of variance by the method of the general linear model (ANOVA). For the variables in which ANOVA found significant differences (*p* < 0.05) between the groups, Bonferroni *post-hoc* analysis was used for multiple comparisons. Repeated-measures ANOVA was used to examine the effects of the biomechanical constraints of the HLJ task on RT (angle: medial and lateral; view: dorsal and palmar; hand: right and left). Significant results were analyzed with the *t*-test for paired samples. Finally, to determine if age predicts efficiency in the MI task, a simple regression analysis was performed.

## Results

As shown in Table 1, all five groups had a similar representation of both sexes (χ^2^ = 0.533; *p* = 0.137). Nine children were excluded for being left-handed and five were excluded for not being able to discriminate right and left.

### Effects of Biomechanical Constraints

#### Medial Rotation vs. Lateral Rotation

**Figure 2:**
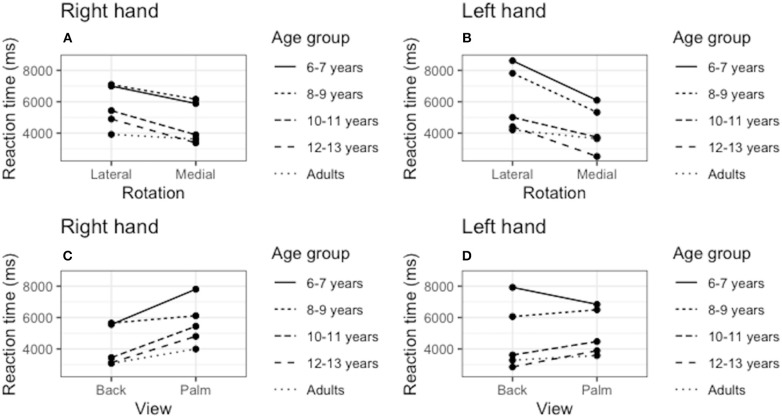
Effect of biomechanical constraints. We verified the reaction time (RT) averages to judge the stimuli in medial rotations compared to ‘lateral rotations for the right hand **(A)** and left hand **(B)**. We also compared the RT to judge the stimuli presented in the palmar and dorsal views for both the right **(C)** and left **(D)** hands.

Figure 2 shows the presence of the effect of biomechanical constraints, as indicated by ANOVA showing a significant interaction between the rotation angle and RT [*F*_(4, 180)_ = 29.61; *p* < 0.006; η^2^ = 0.580]. Participants were faster to judge the stimuli presented in medial than in lateral rotations (*p* < 0.05). Bonferroni's comparison showed that all age groups were faster to judge medial rotations for both right hand stimuli (6–7 years old: *p* = 0.001, d = 2.06; 8–9 years old: *p* < 0.000, d = 1.82; 10–11 years old: *p* < 0.001, d = 1.64; 12–13 years old: *p* < 0.013, d = 1.12; adult: *p* < 0.026, d = 0.98), and left hand stimuli (6–7 years old: *p* < 0.001, d = 2.06; 8–9 years old: *p* < 0.000, d = 1.95; 10–11 years old: *p* < 0.001, d = 1.16; 12–13 years old: *p* < 0.013, d = 0.81; adult: *p* < 0.026, d = 0.86). We also found a significant interaction between age and RT [*F*_(4, 180)_ = 29.61; *p* < 0.006; η^2^ = 0.580], with the groups 6–7 and 8–9 years of significantly slower than the groups 10–11, 12–13 years, and adults (*p* < 0.05). The other comparisons between the groups did not result in statistically significant differences (Figure 2).

#### Dorsal View vs. Palm View

As shown in Figure 2, the ANOVA showed a significant interaction between the rotation angle and the stimulus view [*F*_(4, 180)_ = 12.81; *p* < 0.001; η^2^ = 0.346]. Children of the group 6–7 years were only faster to judge dorsal view stimuli for right-hand figures (*p* < 0.001, d = 0.68). The opposite was observed for the left hand, as lower RTs were observed for the palm view (*p* < 0.001, d = −0.22). Children of the group 8–9 years did not show significant differences to judge back and palm stimuli [*F*_(4, 180)_ = 2.05; *p* = 0.161; η^2^ = 0.060]. Pairwise comparisons showed that groups 10–11, 12–13 years, and adult were faster to judge hand laterality presented in back view, both for the stimuli of the right hand (10–11 years old: *p* < 0.001, d = 0.96; 12–13 years old: *p* < 0.013, d = 0.98; adult: *p* < 0.026, d = 1.58), and left hand (10–11 years old: *p* < 0.001, d = 0.80; 12–13 years old: *p* < 0.001, d = 0.54; adult: *p* < 0.001, d = 0.86).

### Age Differences

A simple regression analysis revealed that age is a significant correlate of performance in the MI task in terms of accuracy (*r*^2^ = 0.357; β = −0.605; *t* = −6.357; *p* < 0.001) and RT (*r*^2^ = 0.329; β = −0.582; *t* = −5.982; *p* < 0.001).

#### Accuracy

**Figure 3:**
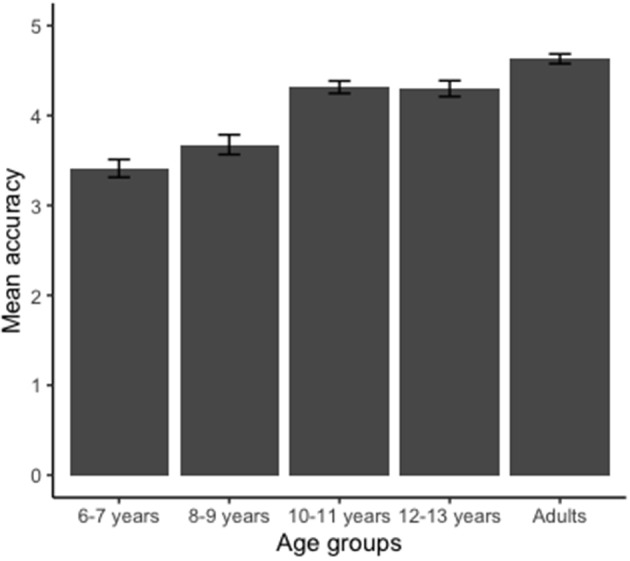
Mean values obtained for the accuracy of the hand laterality judgment task. Groups 6–7 and 8–9 years were less accurate than groups 10–11, 12–13 years, and adult (*p* < 0.001). Error bars indicate standard error.

The average of the correct answers (accuracy) is shown in Figure 3. First we confirmed that all participants indeed involved in MI to solve the task by detecting if they responded better than chance (with hit rates above 50%). Accuracy analysis revealed a major group effect [*F*_(4, 180)_ = 17.560; *p* < 0.00; η^2^ = 3.79]. The groups 6–7 and 8–9 years were significantly less accurate than the groups 10–11, 12–13 years, and adult group (*p* < 0.05). Groups 6–7 and 8–9 years responded similarly (*p* > 0.05). In addition, the groups 10–11, 12–13 years, and adult responded similarly in terms of accuracy (*p* > 0.05).

#### Reaction Time

**Figure 4:**
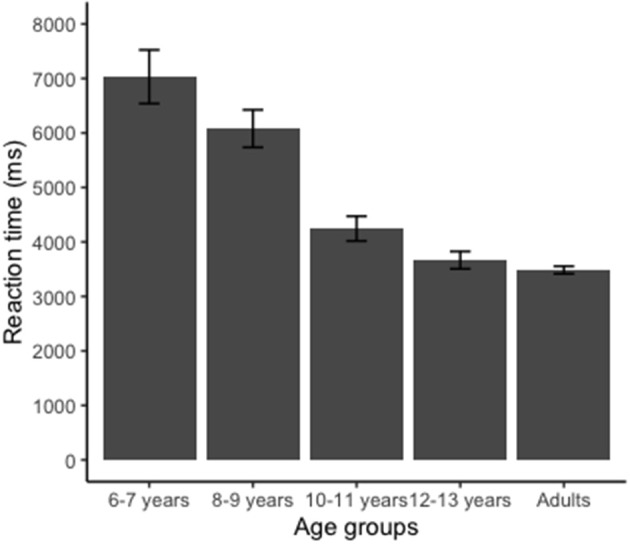
Reaction time (RT) for the hand laterality judgment task. Group 6–7 years presented longer RT than groups 8–9, 10–11, 12–13 years, and adult (*p* < 0.001). Group 8–9 years showed longer RT than groups 10–11, 12–13 years, and adult (*p* < 0.001). Error bars indicate standard error.

Figure 4 shows the mean RTs for the five age groups in the HLJ task. ANOVA identified a significant effect on RT [*F*_(4, 180)_ = 17.5; *p* < 0.001, η^2^ = 0.615]. Analysis with Bonferroni showed that the youngest group (6–7 years) was significantly slower than the other groups (*p* < 0.05). Group 8–9 years was also slower than the groups 10–11, 12–13 years, and adult. We also found that the adult group did not differ regarding the RT when compared to the older children groups (groups 10–11 and 12–13 years).

## Discussion

Our results revealed that the youngest children studied (group 6–7 years) were able to perform MI to solve the mental rotation task. There was a biomechanical restriction effect for all age groups, with all presenting lower RT to recognize the stimuli oriented in medial positions than the stimuli of lateral orientation. We also found that when task stimuli were presented in the dorsal view, the volunteers had lower RT to judge the stimuli. Finally, we observed a progressive improvement in the performance of the task as the age of the participants increased, reaching stabilization after 10 years, when the performance in the HLJ task was similar to that of the adult group.

The age at which children begin performing HLJ tasks using MI is not sufficiently clear in the literature. This is partly because the HLJ task is cognitively complex as it depends on the ability to mentally rotate images, on the ability to discriminate right and left, and on the ability to integrate visual and proprioceptive afferences. Several studies suggested that children may perform mental rotations at 5 years of age, albeit at a slower rate than adults (32, 43, 44). According to Belmont and Birch (45), it is expected that from the age of 6 the child will be able to recognize in himself/her right and left limb. Between 5 and 7 years old children acquire the ability to integrate visual and proprioceptive afferences necessary for the execution of movement (21, 46, 47). We found that the youngest children studied herein (group 6–7 years) used MI to solve the mental rotation task, suggesting that at these young ages children already have the cognitive requirements to perform the HLJ task. As our study did not involve children younger than six, the minimum age at which the ability to use MI to solve mental rotation tasks occurs remain an open question. Notwithstanding, our results indicate that because children 6–7 years old are able to use the mental rotation strategy, it is plausible to think that MI-based interventions could be used in this age group. This suggestion is supported by the literature review conducted by Spruijt et al. (20). After analyzing some studies, Spruijt et al. (20) suggest that MI training is a potential and viable method for the rehabilitation of children aged 5 years and older. Some studies involving populations aged 7 to 12 years highlight the potential of MI training in children (48, 49).

The effect of biomechanical restrictions on medial and lateral rotations was observed in all age groups. However, the accuracy is significantly reduced in the groups 6–7 and 8–9 years, and the RTs of these children are higher than those presented by older children and the adult group. Our findings contrast those reported by Spruijt et al. (30) because they found that 6 years old children were not able do not perform MI tasks. This divergence may be due to experimental approaches as these authors measured the timing of the actions imagined and performed, and not the HLJ task used herein. These contrasting results suggest that performance in MI may be task dependent. For Spruijt et al. (30) the mental chronometry paradigm seems to be a conservative measure that may underestimate individuals' ability to use MI. In this study the authors found that not all healthy adult individuals used MI to solve the task. Thus, we believe that when considering the use of MI in pediatric rehabilitation, it is important for the child to make an individualized assessment of MI ability in order to ensure the effectiveness of the technique. Given the divergent results of studies using different tasks, it may be advisable to use multiple tasks to draw more definitive conclusions about children's ability to use MI.

The classic mental rotation task employed in our study has been widely used to evaluate MI (1, 22, 27). In this task the individuals are required to imagine their hand moving to judge the laterality of the stimulus, thereby making the task highly effective to assess motor information during the mental transformation of hand stimuli (1). This is based on the hypothesis that the effect of biomechanical constraints is indicative of the use of the mental rotation strategy. Thus, the easiest physically executed stimuli are also judged faster supporting the idea that the same biomechanical factors that constrain actual movements also determine imagined movements (50). For Parsons (25), presence of biomechanical effects provides clear evidence that MI has been used to solve the HLJ task.

Additional evidence for the use of the mental rotation strategy comes from the effects of the posture in which the hand was presented. Participants in our study recognized faster stimuli presented in dorsal view. Similarly, Butson et al. (22) reported that children from 5 to 12 years old also presented lower RTs for dorsal view stimuli. Knowing that, to judge stimuli, individuals imagine their hand moving from the current position, a possible explanation for this finding would be that individuals remain with their hands in the dorsal posture while performing the task. Strengthening this hypothesis, previous studies suggested that the time to judge hand laterality is strongly influenced by the member's actual position during task resolution (19, 25). Therefore, in judging the laterality of hand figures, volunteers simulate the movement of their own body from its present (egocentric) position, rather than from an allocentric representation. Shenton et al. (51) evaluated the influence of hand posture on the HLJ task, performing two judgment blocks: one with hands in dorsal posture and a second with hands in palmar posture. There were no significant differences in RT to judge the stimuli, indicating that hand posture during task resolution influences the RT spent to judge the stimuli. These observations suggest that, by recognizing still images of hands in varying positions, subjects move their own hands to their respective positions to arrive at a laterality decision.

In our study, only children from 10 years of age had the facilitating effect of dorsal vision to solve the HLJ task. One possibility is that the recognition of stimuli in dorsal vision represents a maturational effect on the HLJ task. Individuals tend to judge hand stimuli from their current position rather than from a fixed representation in the brain. We believe that the absence of this effect in younger children is due to the fact that, at this age, children did not go through the complete maturation of motor and cognitive processes involved in MI. According to Casey et al. (52), children show increasingly specialized motor and perceptual behavior. This is due to the fact that neural networks become increasingly differentiated with development. For these authors (52), these changes allow older children to process information faster and more accurately than younger children.

The effect of the presented hand posture is modulated by age. Groups involving children aged 10 and older find easier to judge laterality from the dorsal view. A possible explanation for this interaction may be the effect of visual influences. If the mental rotation strategy is used to decide on laterality from an egocentric perspective, the dorsal view is privileged. This effect may take a few years to develop depending on the individuals' experience. This interpretation is supported by evidence indicating visual influences on body schema as shown in the rubber hand experiment (51).

We hypothesized that there would be changes in MI ability as age increased. Our results support this hypothesis by showing progressive improvement in the performance of the HLJ task as the participants' age increased. It is important to highlight, however, that the improvement in motor imaging performance occurred in children up to 10 years old. From that age, performance was similar to that of adults. In line with our results, most studies using the HLJ paradigm also reported increased motor involvement with age (1, 20, 21, 39). The study by Caeyenberghs et al. (21) compared the performance in MI through the HLJ task of 7 and 8 year old, 9 and 10 years old, and 11 and 12 years old. The results showed that older children were faster and more accurate than younger children, suggesting changes in MI as they age. Strengthening this hypothesis, the articles on age-related differences in MI analyzed in the Spruijt et al. (20) review indicate that children's ability to perform the task accurately increases with age.

Indeed, from 10 years old, the performance in the HLJ task resembled that of the adult group. We also found a progressive decrease in RT as participants' age range increased. Children of 6–7 years old were slower than those of the other age groups and children aged 8–9 years were also slower when compared to older age groups. Indeed, the performance in the HLJ task of children aged 10 and older was similar to that of adults. We found that the adult performance level with regards to accuracy and RTs is reached when children reached 10 years of age. This result probably reflects the maturation of the brain areas (posterior parietal cortex, premotor area, cerebellum, and frontoparietal region) involved with the mental simulation of body part movements (21, 22, 53).

Our results point to an improvement in MI capacity as age increases. Similar results were also found by Caeyenberghs et al. (21). This improvement in MI as age is supported by the development and maturation of a set of complex cognitive processes (21). Significant structural and functional changes occur in the child's brain during childhood. According to Casey et al. (52) children show increasingly specialized motor and perceptual behavior due to the fact that neural networks become increasingly differentiated with development. For these authors, these changes allow older children to process information faster and more accurately than younger children. Casey et al. (52) further state that fronto-parietal coupling is greatly increased throughout childhood, in particular between 6 and 10 years of age. This explains why the children in our study showed progressive improvements in performance with age, as well as a similar response pattern to adults when they reached the age of 10 years.

Our results point to a non-linear improvement in RT, corroborating the findings of Fuelscher et al. (38). We found that the ability of MI progressively improves until 10 years of age, after that age, the performance is similar to that observed in adults. Thus, as in previous studies (21, 38), our study points to a substantial maturation in MI ability in the early years of elementary school, becoming mature in late childhood and early adolescence.

For Fuelscher et al. (38) there is evidence that the development of MI can also be influenced by the development of general cognitive factors, such as the visuospatial capacity of working memory. Indeed, these interindividual differences in MI ability can be explained by cognitive and motor skills that may facilitate or restrict the development of MI. Previous studies suggest that executive functioning, planning ability, movement experience, working memory, and intelligence may all influence MI (20, 21, 54, 55). Nonetheless, we suggest that MI is a continuous and progressive refinement throughout childhood and early adolescence, becoming progressively stronger with advancing age. We attribute the maturation in MI capacity to the development of neural networks linked to the internal simulation of movements. This maturation in the ability to perform imagined movements can be interpreted in terms of a general development of the cognitive processes involved in motor representation. This development is mainly determined by internal changes in the structures of the prefrontal and parietal cortex (56). This is in line with previous evidence that the parietal cortex is involved in the formulation of internal models associated with motor imagery and the internal representation of action (56). Vargas et al. (57) also point out that the evolution of MI in children is also related to the maturation of the supplementary motor area, premotor area, primary motor cortex, basal ganglia and cerebellum.

### Limitations and Implications

Our results provided evidence that children aged 6 years and older are able to use MI to solve the mental rotation task. However, as our study did not involve children under 6 years old, the minimum age at which this ability is present remains an open question, which is a limitation of this study. With a sample composed of ages ranging from 6 to 13 years, our results suggest that there is a progressive improvement in MI as age increases. These results are in line with previous studies (1, 20, 21, 39). However, it is not yet possible to make definitive inferences about the exact trajectory of development. For this, studies with longitudinal methodological design would be necessary.

Due to the characteristics of the MI skill, we believe the divergent results are due in part to the use of different tasks. In addition, individual differences may also influence this ability, such as cognitive functioning. Studies suggest that working memory, attention, planning, and intelligence may facilitate or restrict the development of MI (20, 21, 54, 55). According to previous studies, motor planning ability and motor skills may also influence MI performance (37, 38). However, our methodological design did not include measures to assess these skills, which is one of the limitations of the present study. Thus, experiments that evaluate the development of MI controlling cognitive and motor skills are still a challenge for future studies.

The use of motor imagery by children has important theoretical implications. Recent studies suggest that performing MI activates specific sensorimotor representations involved in the planning and execution of motor acts (58). Thus, MI is a useful tool in pediatric rehabilitation. Few studies have investigated the use of MI in the rehabilitation of children. Buccino et al. (59) applied MI training by observing action associated with real movements in children with cerebral palsy and found beneficial results. In this experiment, the authors observed that the group of children who watched other people's videos producing actions led to an increase in motor function, which was not observed in children who watched videos without motor content. One advantage of the implicit use of MI by observing the action is that it can be beneficial for small children who cannot be educated on the use of MI. Our results provide contributions about the development of MI in children setting an important starting point for future research interested in assessing the effectiveness of MI as a tool for pediatric rehabilitation.

## Conclusion

The use of the mental rotation strategy by 6–7 year-olds has important theoretical implications and further investigation of the neuro-cognitive foundations is warranted. The results obtained herein indicating the influence of biomechanical restrictions and hand posture suggest that children use the strategy of mental representation of the body part. Future research needs to clarify the role played by hand laterality judgment, mental object rotation, and cognitive control processes in HLJ execution. Our results also have important clinical applications. There is currently a strong interest in the use of MI-based interventions for the development and rehabilitation of cognitive and motor functions and the results presented herein indicate that this strategy may be used in children as young as six.

## Data Availability Statement

The datasets generated for this study are available on request to the corresponding author.

## Ethics Statement

The studies involving human participants were reviewed and approved by Research Ethics Committee of the Federal University of Minas Gerais (COEP/UFMG). Written informed consent to participate in this study was provided by the participants' legal guardian/next of kin.

## Author Contributions

DS and TC delineated the study. PF, RB, and VH conducted the neuropsychological evaluation. All authors contributed in analyzing the results and writing the paper. All authors read the final version of the paper and agree with the content of the manuscript.

### Conflict of Interest

The authors declare that the research was conducted in the absence of any commercial or financial relationships that could be construed as a potential conflict of interest.
